# Salidroside as a Novel Protective Agent to Improve Red Blood Cell Cryopreservation

**DOI:** 10.1371/journal.pone.0162748

**Published:** 2016-09-15

**Authors:** Noha A. S. Alotaibi, Nigel K. H. Slater, Hassan Rahmoune

**Affiliations:** 1 Department of Chemical Engineering & Biotechnology, University of Cambridge, CB2 3RA Cambridge, United Kingdom; 2 King Abdulaziz City for Science and Technology Kingdom of Saudi Arabia P.O Box 6086, Riyadh 11442, Saudi Arabia; Universidade Nova de Lisboa Instituto de Higiene e Medicina Tropical, PORTUGAL

## Abstract

Glycerol and trehalose have been widely examined as protective agents in the cryopreservation of red blood cells (RBCs). However, the effectiveness of these reagents alone on cell viability is moderate. Here, the addition of salidroside attenuated oxidative damage of sheep RBCs prior to and post cryostorage. The supplementation of salidroside to the cryopreservation media containing 10% glycerol improved RBC survival by approximately 61.1±4.8% vs 37.9±4.6%. A smaller effect was seen in RBCs cryopreserved in 300 mM trehalose where the addition of salidroside improved survival by 7.6±0.3%. Furthermore, the addition of salidroside to cold storage solution demonstrated a significant reduction of haemolysis after 4 days for RBCs loaded with either glycerol or trehalose, compared to cells incubated without salidroside. RBCs survival was 2-fold greater following freezing in trehalose, compared with glycerol. After 10 days, salidroside enabled a lower haemolysis of 16.7±1.3% compared to 29.0±8.4% for cells incubated without salidroside. However, salidroside had no effect on RBCs which had been frozen in glycerol as the resulting haemolysis rate by day 10 was approximately 60%. Salidroside increased glutathione reductase activity and decreased lactate dehydrogenase activity. Furthermore, it led to reduced carbonylation of proteins in both glycerol and trehalose loaded cells. Finally, no effect on lipid peroxidation was found in the glycerol loaded RBCs although this was reduced in RBCs loaded with trehalose and salidroside. The present findings confirm the potential use of salidroside as a novel protective agent in cryopreservation and refrigerated storage of sheep RBCs.

## Introduction

Red blood cell (RBCs) storage *ex-vivo* is important for patient care worldwide. Lost blood must be replaced in major surgeries and RBCs deficiencies must be countered in the treatment of health conditions such as anaemia and sickle-cell diseases. To meet these demands, blood banks are now commonplace which use refrigerated storage methods to preserve blood or RBCs. However, this approach only allows blood storage for a maximum of 42 days. The quality of stored RBCs becomes compromised with prolonged storage time [1], rendering these unsuitable for clinical transfusion [2]. In addition, RBCs quality and survival rate post transfusion may be compromised regardless of storage times [2]. Molecular signatures associated with RBCs loss of functional properties during cold storage have been identified by proteomic [3], lipidomic [4] and metabolomic [5] approaches. These investigations have all shown that oxidative stress pathways may be activated during the cold storage and freeze-thaw processes. Thus, studies attempting to optimize the conditions of RBCs cryopreservation are of major importance in light of responding to the current pressing clinical needs.

Due to ice crystal formation at low temperatures, RBCs experience mechanical traumas and adverse osmotic changes, which lead to oxidative stress and then haemolysis [6]. Therefore, cryoprotective agents (CPAs) are used in attempts to minimize the structural and biological damage during the freeze-thaw process [7]. There is wide range of CPAs used for RBCs cryopreservation but the only compound that has been clinically licensed for this purpose is glycerol. Glycerol is a cell-permeable compound and is used at a 1–2 M concentration for blood cell cryopreservation (20%—40% ratio, glycerol—blood) [8]. Cryopreservation of RBCs in glycerol is achieved using either high concentrations of glycerol and slow freezing at -80°C or low glycerol concentrations and fast freezing in liquid nitrogen. The post-thawing storage time at 2–6°C for RBCs frozen in high glycerol concentrations can be up to 7 days [9] while low glycerol concentrations result in post-thawing storage times of only 24 hours [10]. In addition, removal of glycerol must be performed prior to transfusion using a series of washing steps and this can lead to cell loss, as well as being expensive [11] and time consuming [12]. As a consequence, this process does not offer immediate access to blood and can thus be a limitation in blood transfusion emergency situations requiring more rapid access.

Trehalose is known as a natural CPA and offers cellular protection against external stresses such as desiccation and dehydration [13]. Trehalose has been used for freeze storage of mammalian cells, including RBCs [14]. Studies have shown that the trehalose cryoprotective mechanism involves promotion of vitrification, biomolecule stabilization, prevention of ice crystal formation and consequential osmotic alterations during freezing [15, 16, 17]. The RBC phospholipid bilayer has a low permeability to trehalose under normal conditions and research efforts have focused on developing methods to deliver this carbohydrate-based compound into cells such as liposome loading, electroporation or membrane permeabilization using polymers such as PP-50. The later has led to the successful loading of trehalose into RBCs, resulting in 60 to 80% cryosurvival rates [14].

Despite the protective role of CPAs, freeze-thaw processes can still cause cellular changes leading to oxidative stress. This can result in serious cellular damage via increased production of free radical oxygen species [18]. In addition, recent studies have suggested that trehalose may have an oxidative effect on RBCs via osmotic shock despite its ice crystal inhibiting property [19]. Thus, optimizing RBCs cold storage and cryopreservation processes to minimize structural and functional damage is an important area of research.

In the present study, we have assessed the effects of a novel CPA called salidroside (Sal) on sheep RBC biological functions before and after cryopreservation using glycerol and the trehalose/PP-50 combination. Sal is a tyrosol glucoside and the active component of the herb *Rhodiola rosea*, which has been used to prevent high altitude sickness [20]. It has also been suggested to act as antioxidant through the observation that it can protect human RBCs against hydrogen peroxide-induced pro-apoptotic effects [21].

## Materials and Experimental Design

### Materials

Defibrinated sheep RBCs were purchased from TSC Bioscience Ltd (Buckingham, UK). Dihydrate trehalose, salidroside, sterilised filtered dulbecco’s phosphate buffer saline (DPBS), sodium chloride, glucose, adenine, mannitol, Drabkin’s reagents and glycerol were purchased from Sigma-Aldrich (Poole, UK). The membrane synthetic permeabiliser PP-50 was synthesised in-house, as described previously^22^. Glutathione reductase, lactate dehydrogenase and lipid peroxidation (MDA) assay kits were purchased from Abcam (Cambridge, UK) and the protein carbonyl colorimetric assay kit was purchased from Cayman Chemical Company (Ann Arbor, MI, USA). The Annexin-V apoptosis detection kit was purchased from BD Pharmingen (Oxford, UK).

### Experimental design

The study was divided into three phases. In the first phase, 1 ml of 15% haemtocrit RBCs were prepared and incubated in either PBS, PBS combined with 200 μM Sal, 300mM trehalose plus 100 μg/mL of the polymer PP-50 or trehalose plus PP-50 plus 200 μM Sal using eppendorf tubes. The concentration of Sal was optimised at the above concentration by titration (S1A Fig**)**. In the second phase, RBCs were frozen in liquid nitrogen and thawed 24 hours later. In the third phase, 10% glycerol was removed from RBCs post thawing and washed cell were stored at 4°C for up to 10 days in sodium-adenine-glucose and mannitol (SAGM) solution with and without Sal.

### Solutions preparation

Trehalose (300 mM) was made in DPBS. Then, the polymer PP-50 was added to a final concentration of 100 μg/mL for trehalose loading. 10% Glycerol was, shown to possess similar protective effect on RBCs to normally used 20% glycerol, prepared in DPBS. In addition, 10% Glycerol use requires less aggressive RBCs washing steps post RBCs thaw. SAGM was prepared by adding 8.77 grams sodium chloride, 9.0 grams glucose, 0.169 grams adenine and 5.25 grams mannitol in 1000 mL sterilise water. Salidroside (200 μM) was added as required to the solutions.

### RBCs preparation

Defibrinated sheep RBCs obtained from TSC Bioscience were pelleted by centrifugation at 10,000 x g for 3 minutes. The supernatants were removed and the pellets washed three times with PBS. Then, pellets were suspended in 1 mL of 300 mM trehalose plus 100 μg/mL PP-50 to yield 15% RBCs hematocrit, as described previously^22^ with or without 200 μM salidroside. For the glycerol freezing solution, RBCs were suspended in 10% glycerol in DPBS with and without 200 μM salidroside. The RBCs were incubated for 2 hours in the above solutions and pelleted by centrifugation as above to measure haemolysis (S1B Fig).

### Haemolysis measurement

The RBCs haemoglobin (Hb) content was suspended in Drabkin’s reagent for total Hb measurement while free Hb was measured in supernatants post centrifugation at 10,000 x g for 3 minutes. The supernatants were collected and mixed with Drabkin’s reagent and the mixtures were incubated 20 minutes at room temperature. The absorbance of each sample was measured at 540 nm with a Spectrostar Nano plate reader (BMG Labtech; Aylesbury, UK). The percentage of haemolysis was calculated as follows: Supernatant OD 540nm / total haemoglobin OD540nm x 100%.

### Cryopreservation and cryosurvival measurement

RBCs pellets were re-suspended in fresh solutions containing different combinations of trehalose, glycerol and Sal then submerged in liquid nitrogen for 24 hour. The RBCs were thawed at 37°C in a water bath and haemolysis was measured as described above for survival assessments.

### RBC haemolysis after thawing

Post-thawing, RBCs were centrifuged at 10,000 x g for 3 minutes and washed twice in DPBS. The cell pellets were re-suspended in SAGM with and without Sal. As part of the recovery period, RBCs suspensions were stored at 4°C and haemolysis was assessed every two days for up to 10 days.

### Biochemical assays

Enzymatic activities assessments were performed using 1 x 10^7^ RBCs before and after thawing according to the manufacturer’s instructions. The glutathione reductase (GR) assay is based on measuring spectrophotometrically the resulting chromophore [TNB] (e. g. Sulfhydryl-glutathione and 5,5’-dithiobis (2-nitrobenzoic acid) [DNTB] level at 405 nm. The first and second readouts were measured after 5 and 10 minute intervals using the Spectrostar Nano plate reader.

The lactate dehydrogenase (LDH) assay was performed according to the manufacturer’s instructions. The quantity of NADH was detected spectrophotometrically at 450 nm by mixing NADH detection buffer with the cell supernatant and lysate. The first readout being taken immediately and the samples incubated in the dark at 37°C with a final reading at 30 minutes.

### Protein and lipid oxidation assays

Post thaw, RBCs protein oxidation was assessed using the carbonylation assay according to the manufacturer’s instructions. Briefly, a reaction between 2,4-dinitrophenylhydrazine (DNPH) and oxidized carbonyl groups on proteins was conducted using Cayman’s protein assay kit. The derivatized carbonyl groups were quantitated by reading spectrophotometrically at 375 nm.

Lipid peroxidation was determined using 10^6^ cells/mL RBCs lysates by the formation of malondialdehyde-thiobarbituric acid (MDA-TBA) adduct in acidic condition at 95°C for an hour. The absorbance of the samples was measured at 532 nm using the Spectrostar nano plate reader following the manufacturer’s instructions. The MDA concentration was expressed in nmol.

### Statistical analysis

All experiments were performed in three biological replicates. Results were presented as mean ± standard deviation. Significance differences between groups were determined using Student’s t-test for paired and unpaired observations. *P* values <0.05 were considered significant.

## Results

### Sal effect on haemolysis post-incubation and survival of RBCs frozen in Glycerol +/- Sal versus Trehalose +/- Sal

**Fig 1:**
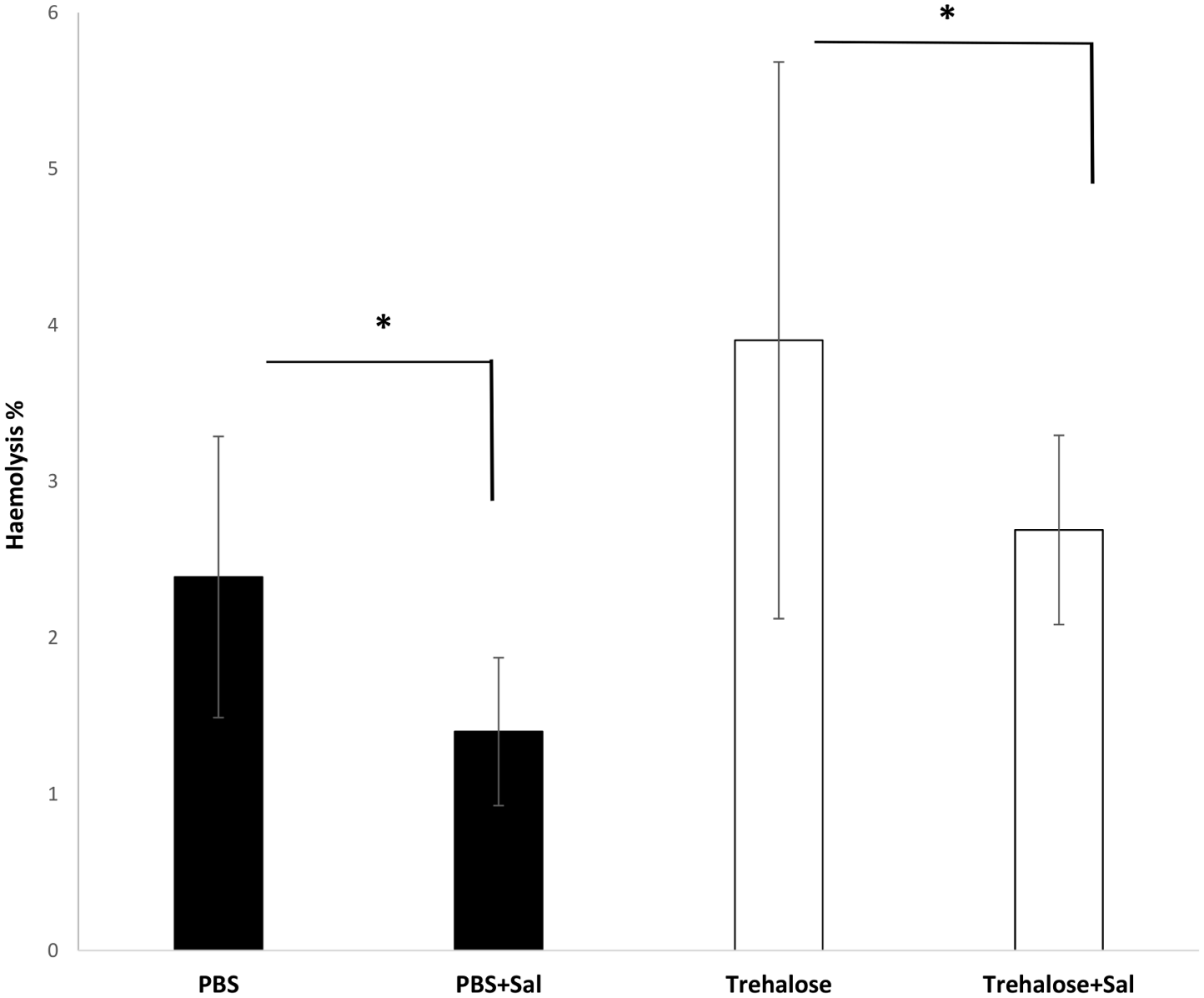
Prior freezing sheep RBCs, induced haemolysis was measured after 9 hours RBCs incubation at 37°C in PBS or trehalose/PP-50 loading with and without Sal. The data are expressed as mean ± SD (p <0.05).

Prior freezing sheep RBCs, the effect of Sal on haemolysis rate during trehalose loading at 37°C was investigated using Drabkin’s reagent. The results show that during the incubation conditions, essential preparation step prior freezing, trehalose triggered haemolysis (Fig 1) and has no effect on RBCs GR and LDH activities (S1C Fig). The haemolysis that occurred in RBCs incubated in PBS containing 200 μM Sal was minimal 1.4±0.4% whereas this was higher in RBCs incubated in PBS alone at 2.4±0.9%. Prior freezing sheep RBCs, Sal also showed a protective effect in RBCs incubated in trehalose/PP-50 with haemolysis occurring for 2.7±0.6% of the cells, compared to 3.9±1.8% for cells incubated in trehalose/PP-50 without Sal. Although the data showed there is a trend toward less haemolysis in the presence of Sal in the cryomedia prior RBCs freezing step, the induced haemolysis rate was below 4% under all conditions and was therefore not significant in any case.

### Effect of Sal on RBC survival post thawing

**Fig 2:**
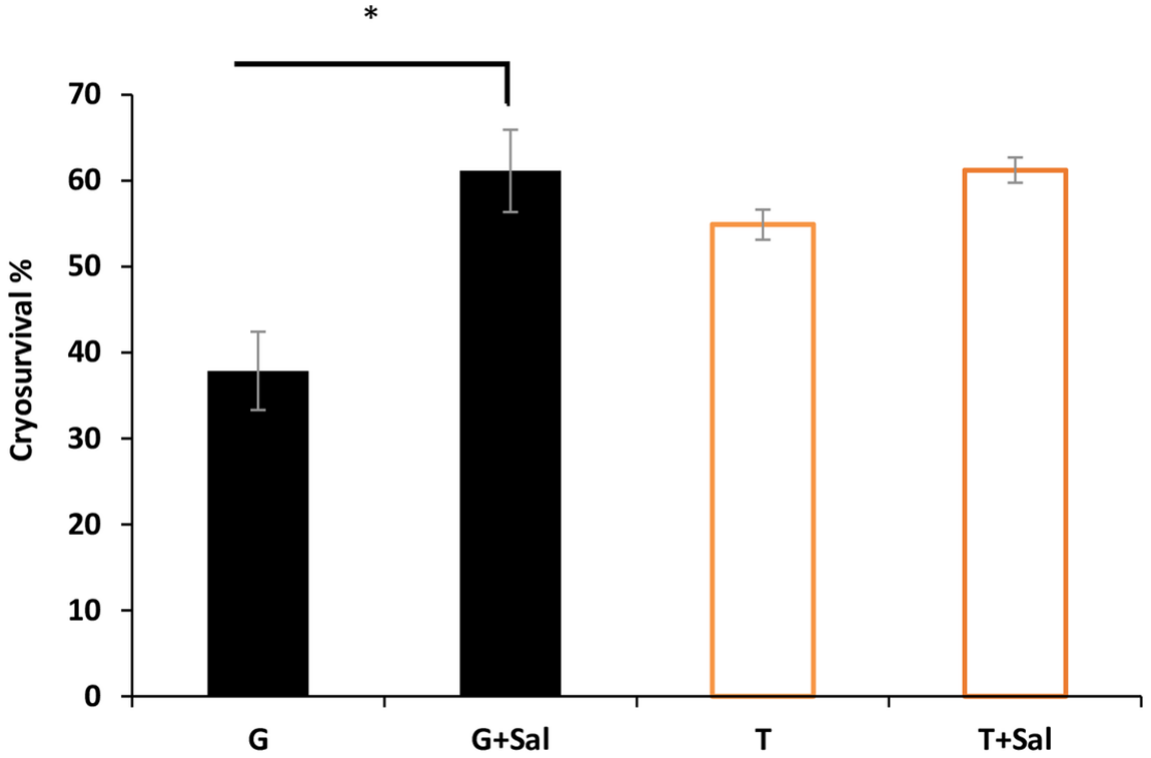
Effect of Sal on RBCs cryosurvival. RBCs were frozen in either glycerol (G) or trehalose (T) without or with Sal (G+Sal, T+Sal). After thawing, RBCs were washed and placed in SAGM media for cold storage and their survival rate was measured 24 hours later using the haemolysis assay. Data are expressed in mean ±SD and * indicates a significant difference (p<0.05).

The effect of Sal addition in the cryomedia was assessed 24 hours after thawing by measuring the percent of haemolysed cells versus the survival using Drabkin’s assay (Fig 2). Sheep RBCs frozen in 10% glycerol (G) in the presence of Sal (G+Sal) showed a survival rate (61.1±4.8%) that was approximately 1.6 fold greater than that seen for cells frozen in glycerol (G) alone (37.9±4.6%) (*p* = 0.0001). Cryo-survivability of RBCs frozen in trehalose (T) with Sal (T+Sal) showed a non-significant (*p*>0.05) higher survival (61.2±1.4%) compared to cells frozen in trehalose (T) only (54.8±1.7%).

### The effect of Sal on RBCs survival and stability post thawing and cold storage for 10 days

**Fig 3:**
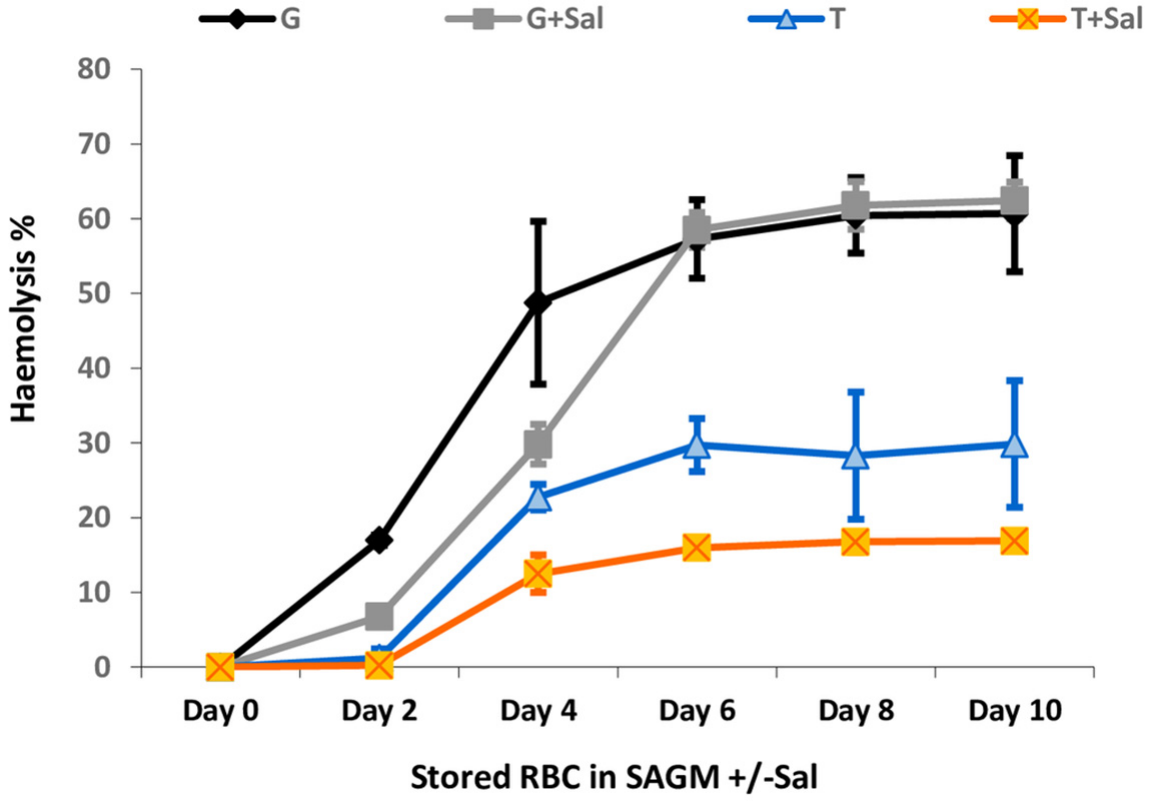
Post-thaw haemolysis rate of RBCs stored and refrigerated in either sodium-adenine-glucose and mannitol (SAGM) solution alone or in the presence of Sal (SAGM+Sal). Survival rate was assessed for up to 10 days. Data are expressed as mean ±SD (p <0.05).

Cryosurvival of RBCs frozen in T+Sal, thawed and stored at 4°C in sodium-adenine-glucose and mannitol (SAGM) with Sal (SAGM+Sal) were assessed at day 10. After thawing, sheep RBCs were washed with PBS to remove haemolysed cells and showed no haemolysis during the first two days of cold storage (Fig 3). By day 4, the measured haemolysis was 12.1±2.5% and this showed a gradual increase over the following days to reach a level of approximately 16.7±1.3% by day 10. RBCs which had been frozen in T alone and stored in SAGM (without Sal) showed a significantly higher haemolysis rate (29±8.4%) compared to the effects seen with Sal at day 4–10 (*p*<0.05).

### Effect of Sal on RBC enzyme activity measurements after 1 and 10 days in cold storage

**Fig 4:**
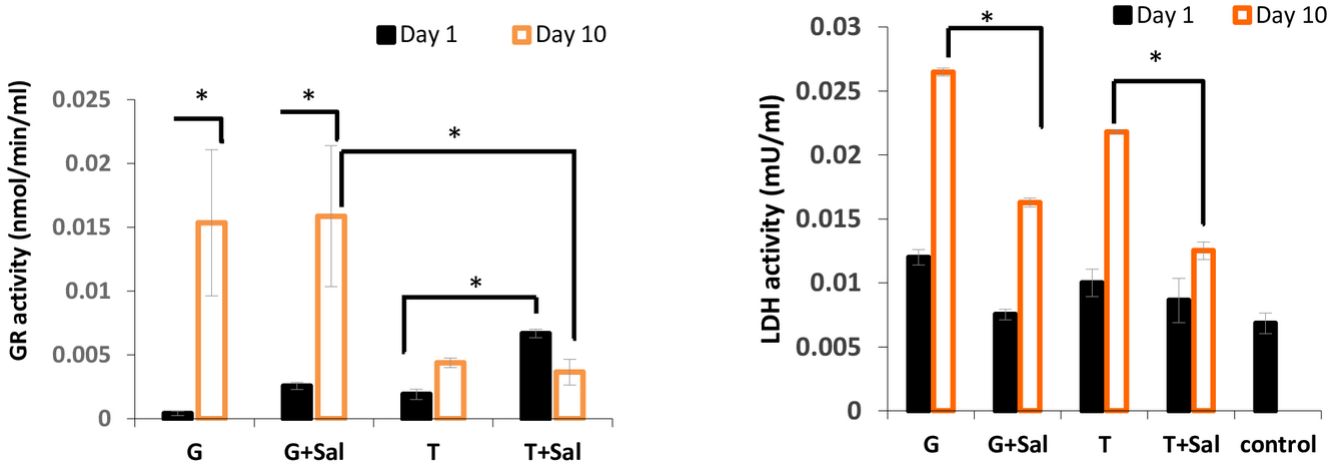
Sheep RBCs enzymatic activities measured after thawing at day 1 and day 10 of cold storage in SAGM media with and without Sal. The graph on the left shows intracellular glutathione reductase (GR) and the graph on the right showed intracellular LDH activity. The data are expressed as mean ± SD (* = p <0.05).

The addition of Sal in the cryomedia prior freezing and post thaw refrigerating media led to an increase in RBCs GR enzymatic activity on day 1 for both the trehalose (0.0066±0.003 vs 0.0004±0.00017nmol/min/mL) and glycerol (0.0025±0.00028 vs 0.0019±0.0004 nmol/min/mL) freezing conditions (Fig 4). On day 10, GR activity increased markedly with Sal addition to the trehalose-frozen RBCs to reach 0.015±0.005 nmol/min/mL, approximately 37 times greater than the activity observed on day 1. In contrast, GR activity in the absence of Sal for the trehalose-frozen cells showed a smaller increase (10-fold) from day 1 to day 10 (0.004±0.003 nmol/min/mL). On day 1, GR activity in RBCs frozen in glycerol and stored without Sal was 0.01199±0.0006 nmol/min/mL and this was higher in the presence of Sal.

The intracellular LDH activity in RBCs was also measured on day 1 and day 10 after thawing. In general, RBCs stored with Sal showed a lower activity of LDH than compared to RBCs stored without Sal. On day 1, the activity of LDH in RBCs was 0.01±0.0006 mU/ml mL and 0.01±0.007 under the glycerol and trehalose freezing conditions, respectively. The LDH activity increased by day 10 to reach 0.026±0.0003 mU/mL and 0.0218±0.0002 mU/mL in the glycerol and trehalose conditions without Sal. Freezing and storage in the presence of Sal on day 1 resulted in a lower level of intracellular LDH activity under the glycerol (0.007±0.0004 mU/ml) and trehalose (0.008±0.002 mU/ml) freezing conditions, respectively. On day 10, LDH activities of the stored RBCs were (0.016±0.0003 mU/mL) and (0.012±0.0006 mu/mL) in glycerol and trehalose survival RBCs respectively (Fig 4).

### RBCs oxidative damage post-storage

**Fig 5:**
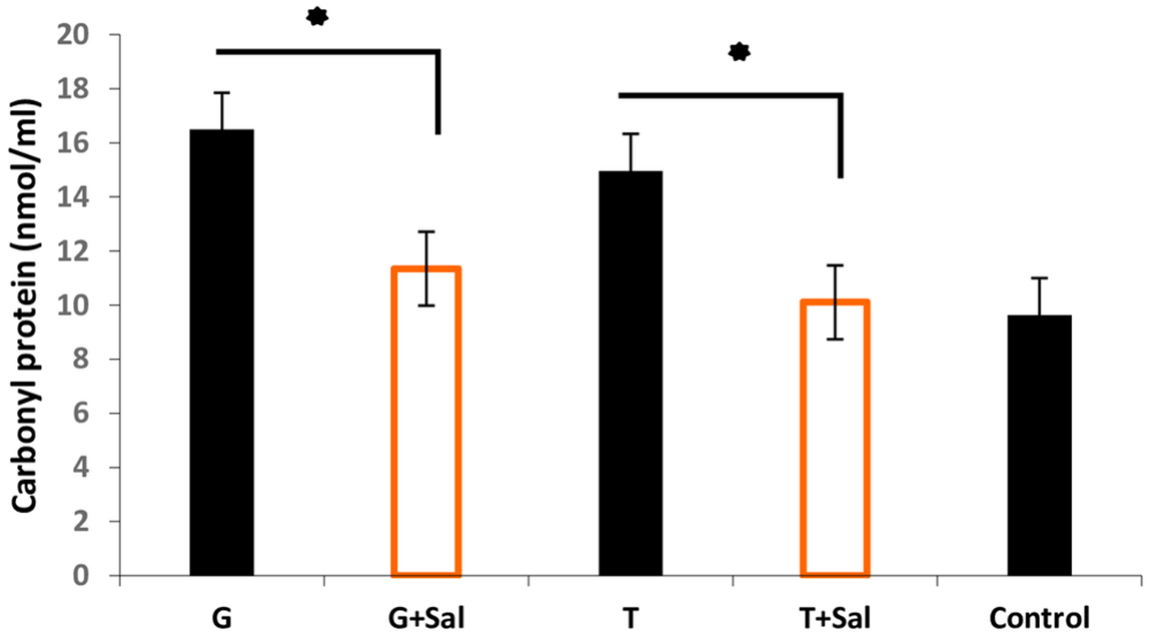
Sal effect on sheep RBCs induced protein carbonylation stored in SAGM+/- Sal post thaw. The protein carbonylation level was determined in RBCs cryopreserved, thawed and stored in SAGM+/-Sal. The control represents protein carbonylation of fresh RBCs not subjected to freeze-thaw. The data are represented as the mean ±SD. (* = p value< 0.05).

We also assessed the protective effect of Sal against the RBCs protein and lipid oxidation post cryopreservation. The results were compared to the control (fresh RBCs not subjected to freeze-thaw) reflecting the protein carbonylation level on day 0 after thawing (Fig 5). This showed that the carbonyl protein levels in RBCs stored in SAGM immediately post thawing were 16.49 nmol/mL and 14.99 nmol/mL for the glycerol (G) and trehalose (T) freezing conditions, respectively. RBCs stored in SAGM with Sal showed lower carbonyl protein levels of 10.1 nmol/mL and 9.63 nmol/mL for the glycerol and trehalose freezing conditions, respectively (*p*<0.05).

**Fig 6:**
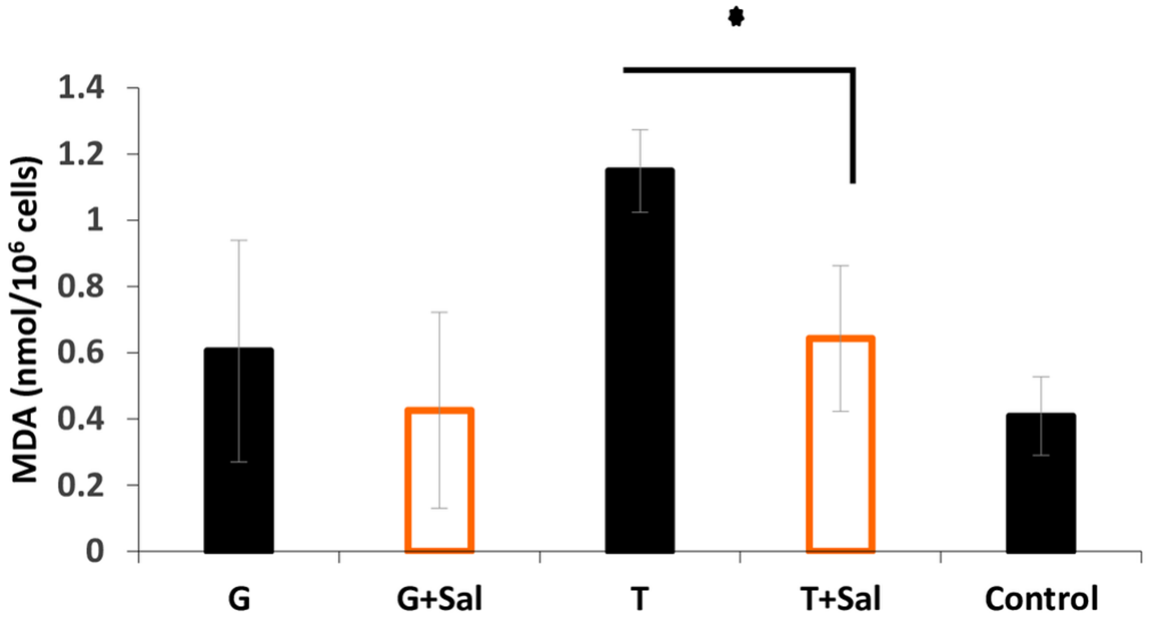
Sal effect on sheep RBC’s lipid oxidation in SAG-M+/- Sal post thaw. The Lipid peroxidation level was determined in RBCs cryopreserved, thawed and stored in SAGM+/-Sal. The control represents lipid peroxidation of fresh RBCs not subjected to freeze-thaw. The data are represented as the mean ±SD. (* = p value< 0.05).

The oxidative damage on RBCs lipids and the effect of Sal was determined by measuring malondialdehyde (MDA) levels (Fig 6). The results demonstrated that glycerol had no effect on lipid peroxidation level in RBCs after thawing and refrigeration (0.4±0.29 vs 0.6±0.22 nmol/10^6^ cells for the fresh RBCs control and glycerol freezing conditions, respectively). In contrast, trehalose induced significant lipid peroxidation (1.14±0.125 nmol/10^6^cells), although this was reduced by Sal (0.64±0.2 nmol/10^6^cells).

## Discussion

Blood and RBCs cryopreservation is a life-saving approach and facilitates rapid access to blood samples in cases of emergency as well as for non-vital purposes. However, the conditions of freeze-thaw in this process are less than ideal, leading to a lower supply of viable material. The use of CPAs such as glycerol and trehalose has been widely investigated on different mammalian cells, including RBCs [14,22], and the negative effects (e.g. osmotic shock, oxidative stress, haemolysis) of using these compounds is well known [23,24]. The detrimental effects on RBCs are not restricted to the cryopreservation process, as these are also commonly observed in refrigerated RBCs. However, the effects of the cryopreservation procedure have not been investigated to the same level as for RBCs cold refrigerated storage conditions. In the case of refrigerated RBCs storage, several attempts have been made to improve conditions by using different additives, such as L-carnosine, spermine, phloretin, glutathione and citric acid [25,26].

In this study, sheep RBCs haemolysis and oxidative status before and after cryopreservation have been assessed in the presence of CPAs such as glycerol and trehalose. Most importantly, to further reduce cellular damage caused by CPAs and other freezing effects, we have investigated the use of the novel cryoprotective agent salidroside as a protectant against oxidative damage to RBCs during storage. Previous studies have shown that RBCs incubated with 800 mM trehalose at 37°C resulted in high osmotic pressure causing oxidative injury via reactive oxygen species production and a pro-apoptosis effect [23]. This was similar to the present findings when measuring the oxidative damage on protein and lipid molecules in RBCs following loading with 300 mM trehalose. Interestingly, both glycerol and trehalose showed the same oxidative damage which was seen as higher levels of carbonylated proteins post thaw and lower levels when Sal was present in the media. In contrast, only trehalose appeared to cause RBCs lipid peroxidation, which was reduced by half in the presence of Sal. The data presented here, reflecting the effect of trehalose on non-nucleated sheep RBCs, differs from a recent study [27], which showed that trehalose had a protective effect on lipids when used on nucleated cells (e. g. bovine calf testicular tissue). Likewise, another study [28] suggested that trehalose possesses anti-inflammatory and anti-oxidative stress properties when used in an induced experimental subarachnoid haemorrhage rather than as a cryoprotectant.

Previous groups have attempted to substitute glycerol with carbohydrate based-CPAs (e.g., hydroxyethyl starch) for RBCs cryopreservation but these compounds appeared to have a damaging effect on membrane-associated cytoskeleton proteins [29]. Comparably, cold storage also has an oxidative effect on haemoglobin and the associated cytoskeleton membrane proteins [30]. These studies indicate the need for a novel cryoprotectant agent that reduces RBCs oxidative damage under both cryopreservation and cold storage conditions.

Recently, the anti-apoptotic/oxidative properties of Salidroside were demonstrated when challenging human RBCs with hydrogen peroxide [21]. Here, Sal was investigated for the first time as a novel cryoprotective agent. It has been shown that fresh RBCs incubated in the presence of Sal had no significant effect on haemolsysis with a trend toward better survival under cryopreservation conditions. The protective effect of Sal was confirmed post freezing since RBCs survival following cryopreservation in 10% glycerol supplemented with Sal showed a double recovery rate (61.1±4.8%) compared to RBCs which had been frozen in 10% glycerol alone.

In this study, we used a lower percentage of glycerol (10%) compared to that commonly used in the clinic (20–40%) to avoid aggressive cell wash during glycerol removal [8]. Nevertheless, it was found that 10% glycerol showed similar cryoprotective effect to the FDA-approved concentration of 20%-40% (data not shown). Interestingly, Sal improved the stability of RBCs during post-thaw storage in the SAGM medium at 4°C. In the presence of Sal, trehalose-loaded RBCs showed a greater stability compared to trehalose or glycerol alone. Despite the advantages of using glycerol as a CPA, it is well known that survival of cryopreserved RBCs in this medium is low at 4°C post-thawing [12]. For example, in case of fast-freezing by direct immersion in liquid nitrogen, glycerolized RBCs do not survive more than 48 hours post-thawing [10]. The present results show that Sal can extend the survivability of RBCs that have been cryopreserved in glycerol from 2 to 4 days. This finding suggests that Sal is an ideal novel additive to improve the performance of the clinically approved CPA glycerol for the cryopreservation of human RBCs.

As a further validation, the mechanisms of action of Sal in limiting RBCs storage-damage was studied. After thawing, the presence of Sal in the cryopreservation and refrigerating media found to reduce RBCs LDH supernatant activity and haemolysis (data not shown), which suggests that Sal enhances RBCs anti-haemolytic and anti-oxidative status, whereas in the absence of Sal intracellular LDH activity and haemolysis increased. The increased level of intracellular LDH in hepatocytes has also been described by Eddie S. group [31] where it was shown to be associated with cytotoxicity. The obtained data demonstrated that glycerol has no effect on RBC’s lipid peroxidation level after thawing and refrigeration (0.4±0.29 vs 0.6±0.22) nmol. On the other hand, trehalose induced lipid peroxidation significantly (1.14±0.125 nmol), whereas Sal reduced the oxidative damage of trehalose loaded RBCs (0.64±0.2 nmol). This is likely to result from the anti-oxidative activity of Sal on RBCs as suggested by previous studies [21]. This possibility is consistent with the observed higher intracellular activity of GR in cells which had been frozen in trehalose and Sal.

Furthermore, we showed that protein and lipid oxidation was reduced by Sal. However, lipid oxidation was only seen in trehalose loaded cells. Finally, the present studies found no effect of Sal on RBC’s apoptosis-like during post thaw cold storage (S1D Fig), suggesting that it did not induce this process. Moreover, the present findings are potentially translatable to human RBCs as previously Sal was shown to possess anti-apoptotic properties [21].

## Conclusion

RBCs survival was enhanced by adding salidroside in the cryomedia and storage media. This appeared to be due to the effects of this compound on reducing RBCs oxidative status. In addition, trehalose used as a CPA caused lipid and protein oxidation and while glycerol had an effect on protein carbonylation only. The induced oxidative damage in protein and lipid by CPAs was reduced to a normal level by using salidroside. Moreover, salidroside markedly reduced the haemolysis in refrigerated RBCs post-thawing in comparison to RBCs stored in the absence of salidroside. The present findings demonstrate the potential benefits of using Sal as an additive protective agent along with existing compounds used in cryopreservation and potentially cold storage of blood and RBCs. This opens up a new avenue of translational research in the fields of blood cell, stem cell, infertility treatment and potentially human tissue cryopreservation.

## Supporting Information

S1 Fig **S1A Fig. This is the S1A Fig Title: Sal dose effect on cryosurvival of RBCs**. This is the S1A Fig legend: Sal dose range effects on cryosurvival RBCs. RBCs were frozen in 10% glycerol containing 50–300μM Sal were analyzed for survival. This showed that 200μM Sal had the highest survival at approximately 92.9±1.2%. The data were generated from triplicate analyses. * = *p*<0.05. **S1B Fig. This is the S1B Fig Title: Optimising the incubation time for Sal cryoprotecting effect on RBCs in a mixture of glycerol or trehalose**. This is the S1B Fig legend: RBCs incubation time in glycerol (left) and trehalose (right) solutions with and without Sal. Both experiments tested at the time points of 2 and 9 hours. Data represent mean ± SD. * = *p*<0.05. **S1C Fig. This is the S1C Fig Title: Enzymatic activities in RBCs during trehalose-loading period**. This is the S1C Fig legend: RBCs enzymatic activities during trehalose loading using the polymer PP-50 over different time intervals (0–9 hours) at 37°C. Enzyme activities were measured in RBCs incubated under the conditions indicated in the figure. The panel on the top shows intracellular GR activity and the bottom panel shows LDH activity. Data were expressed as mean ± SD, ** indicates p< 0.01). **S1D Fig. This is the S1D Fig Title: Flow-cytometric analysis for RBC’s phosphatidylserine (PS)**. This is the S1D Fig legend: Flow cytometric analysis of Annexin-V stained phosphatidylserine (PS) in RBCs after storage at 4°C for 10 days. The top panel shows the effect of SAG-M and incubation length on the PS exposure of RBCs which had been frozen in trehalose alone. The bottom panel shows the effect of SAG-M + Sal on the PS exposure of RBCs incubated and frozen in trehalose +Sal. Sal had no effect on the PS exposure. Therefore, the majority of RBCs under both conditions were viable and non-apoptotic (93.66%).
(DOCX)
