# Supplementary material for: Salidroside as a Novel Protective Agent to Improve Red Blood Cell Cryopreservation
Source: PLoS One. 2016 Sep 15;11(9):e0162748. doi: 10.1371/journal.pone.0162748 (PMC5025239; doi:10.1371/journal.pone.0162748)
Supplement: S1 Fig — S1A Fig. This is the S1A Fig Title: Sal dose effect on cryosurvival of RBCs. This is the S1A Fig legend: Sal dose range effects on cryosurvival RBCs. RBCs were frozen in 10% glycerol containing 50–300μM Sal were analyzed for survival. This showed that 200μM Sal had the highest survival at approximately 92.9±1.2%. The data were generated from triplicate analyses. * = p<0.05. S1B Fig. This is the S1B Fig Title: Optimising the incubation time for Sal cryoprotecting effect on RBCs in a mixture of glycerol or trehalose. This is the S1B Fig legend: RBCs incubation time in glycerol (left) and trehalose (right) solutions with and without Sal. Both experiments tested at the time points of 2 and 9 hours. Data represent mean ± SD. * = p<0.05. S1C Fig. This is the S1C Fig Title: Enzymatic activities in RBCs during trehalose-loading period. This is the S1C Fig legend: RBCs enzymatic activities during trehalose loading using the polymer PP-50 over different time intervals (0–9 hours) at 37°C. Enzyme activities were measured in RBCs incubated under the conditions indicated in the figure. The panel on the top shows intracellular GR activity and the bottom panel shows LDH activity. Data were expressed as mean ± SD, ** indicates p< 0.01). S1D Fig. This is the S1D Fig Title: Flow-cytometric analysis for RBC’s phosphatidylserine (PS). This is the S1D Fig legend: Flow cytometric analysis of Annexin-V stained phosphatidylserine (PS) in RBCs after storage at 4°C for 10 days. The top panel shows the effect of SAG-M and incubation length on the PS exposure of RBCs which had been frozen in trehalose alone. The bottom panel shows the effect of SAG-M + Sal on the PS exposure of RBCs incubated and frozen in trehalose +Sal. Sal had no effect on the PS exposure. Therefore, the majority of RBCs under both conditions were viable and non-apoptotic (93.66%). (DOCX) [file pone.0162748.s001.docx]

**Supplementary data: S1**

**Sal dose effect on cryosurvival of RBCs**

**Fig A.** Sal dose range effects on cryosurvival RBCs. RBCs were frozen in 10% glycerol containing 50-300μM Sal were analyzed for survival. This showed that 200μM Sal had the highest survival at approximately 92.9±1.2%. The data were generated from triplicate analyses. * = - *p*<0.05.

**Optimising the incubation time for Sal cryoprotecting effect on RBCs in a mixture of glycerol or trehalose**

**Fig B.** RBCs incubation time in glycerol (left) and trehalose (right) solutions with and without Sal. Both experiments tested the time points of 2 and 9 hours. Data represent mean ± SD. *p*-<0.05.

**Enzymatic activities in RBCs during trehalose-loading period**

**Fig C.** RBCs enzymatic activities during trehalose loading using the polymer PP-50 over different time intervals (0-9 hours) at 37°C. Enzyme activities were measured in RBCs incubated under the conditions indicated in the figure. The panel on the top shows intracellular GR activity and the bottom panel shows the released LDH activity. Data were expressed as mean ± SD, ** indicates p< 0.01).

**Flow-cytometric analysis for RBC’s phosphatidylserine (PS)**


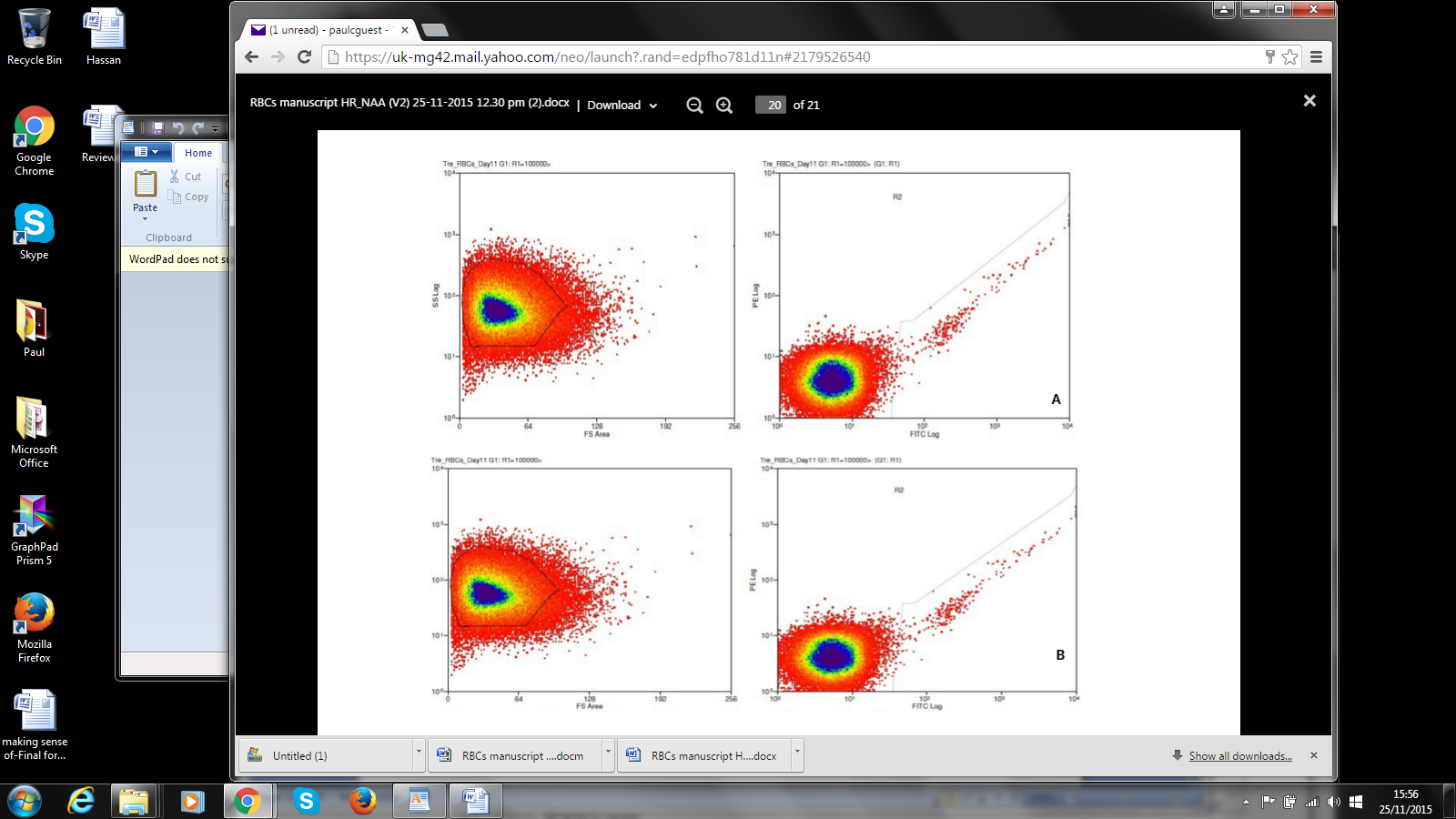


**Fig D.** Flow cytometric analysis of annexin-v stained phosphatidylserine (PS) in RBCs after storage at 4°C for 10 days. The top panel shows the effect of SAG-M and incubation length on the PS exposure of RBCs which had been frozen in trehalose alone. The bottom panel shows the effect of SAG-M + Sal on the PS exposure of RBCs incubated and frozen in trehalose +Sal. Sal had no effect on the PS exposure. Therefore, the majority of RBCs under both conditions were viable and non-apoptotic (93.66%).
